# Computational network pharmacology and molecular simulations to explore the potential mechanisms of *Pyrrosia piloselloides* compounds against prostate cancer

**DOI:** 10.1186/s43046-026-00421-3

**Published:** 2026-10-29

**Authors:** Husnul Havid, Ysrafil Ysrafil, Fatmaria Fatmaria, Muhammad Rifqi Musyaffa, Masira Sony Crespo Sinuhaji, Remi Ayu Pratika

**Affiliations:** 1 Medical Study Program, Faculty of Medicine, Universitas Palangka Raya, University of Palangka Raya, Palangkaraya, 73111 Indonesia; 2 Department of Pharmacotherapy, Faculty of Medicine, Universitas Palangka Raya, University of Palangka Raya, Palangkaraya, 73111 Indonesia; 3 Study Program of Chemistry, Faculty of Mathematics and Natural Sciences, University of Palangka Raya, Palangkaraya, Indonesia

**Keywords:** *Pyrrosia piloselloides*, Network pharmacology, Prostate cancer, Molecular docking, Molecular dynamics

## Abstract

**Background:**

Prostate cancer is major cause of cancer-related morbidity and mortality in men worldwide. Emerging evidence highlights the dragon scale fern (*Pyrrosia piloselloides* or *P. piloselloides*) has demonstrated anticancer potential, but its molecular mechanisms against prostate cancer remain unclear. The study aimed to investigate its potential anticancer compounds and underlying molecular mechanisms using an integrated in silico approach.

**Methods:**

A structured computational workflow integrating network pharmacology, molecular simulations, and MM/GBSA analysis was employed to explore the potential mechanism of bioactive compounds of *P. piloselloides* against prostate cancer-related targets. Additionally, the pharmacokinetic and toxicity profiles were evaluated using ADMET prediction.

**Results:**

Eight bioactive compounds of the plants were identified as potentially interacting with 32 prostate cancer-related proteins, including nine key targets: ESR1, MYC, CTNNB1, PPARG, AKT1, STAT3, BCL2, AR, and ERBB2. Clustering analysis identified 20 proteins within the major cluster, that subsequently dianalisis enrichment idapati bahwa mereka were associated with 235 molecular functions, 142 cellular components, and more than 1,000 biological processes, including epithelial cell proliferation dan identified terlibat dalam 161 enriched pathways, including the PI3K–Akt and ERBB signaling pathways. Docking analysis revealed variable predicted binding affinities, particularly for 7-dehydrodiosgenin 3-acetate against ERBB2 (− 11.85 kcal/mol), AKT1 (− 9.49 kcal/mol), and BCL2 (− 9.38 kcal/mol), stigmasterol against PPARG (− 9.63 kcal/mol), ERBB2 (− 9.94 kcal/mol), and AR (− 9.31 kcal/mol), and 6-hydroxymethaqualone against ESR1 (− 9.34 kcal/mol). Molecular dynamics simulations indicated overall structural stability with localized flexibility, while MM/GBSA analysis yielded favorable predicted binding free energies for ERBB2 with 6-hydroxymethaqualone (− 45.57 kcal/mol), PPARG with stigmasterol (− 41.52 kcal/mol), and BCL2 with 7-dehydrodiosgenin 3-acetate (− 23.35 kcal/mol). The compounds also showed generally favorable predicted ADMET profiles, providing basis for further experimental evaluation.

**Conclusion:**

The integrated computational analysis identified 6-hydroxymethaqualone, stigmasterol, and 7-dehydrodiosgenin 3-acetate as compounds with promising predicted interactions across prostate cancer-related targets. These findings provide a basis for further investigation and experimental validation.

**Supplementary Information:**

The online version contains supplementary material available at https://doi.org/10.1186/s43046-026-00421-3.

## Introduction

Prostate cancer is the second most commonly diagnosed cancer among men worldwide, second only to lung cancer. It is a significant public health concern as the fifth leading cause of cancer-related deaths globally. Risk factors for prostate cancer include diets high in animal saturated fats, red meat, and dairy products, as well as environmental factors, excessive alcohol and coffee consumption, and vitamin D deficiency [1, 2]. The clinical manifestations of prostate cancer greatly affect quality of life, impacting sexual health, physical well-being, and psychosocial aspects [3, 4]. Resistance to chemotherapeutic agents, whether intrinsic or acquired, such as docetaxel and bicalutamide, poses a significant challenge in current prostate cancer treatment strategies [5–7].

Growing evidence, the extracts of *Pyrrosia piloselloides* have demonstrated potential as anticancer therapies. This plant has shown chemoprotective effects against cervical carcinoma cells in vitro using the HeLa cell line, with similar effects observed on breast cancer cells (MCF-7). Additionally, *Pyrrosia* is known to contain phytochemical compounds such as phenolics and flavonoids [8, 9] Derivatives of these compounds, including quercetin, curcumin, anthocyanins, and apigenin, have been shown to inhibit prostate cancer by targeting the androgen receptor axis and exerting epigenetic effects such as modifying histones and regulating microRNA expression [10, 11].

Additionally, the quercetin have been reported to inhibited the proliferation of prostate cancer cells by upregulating caspase-3 and downregulating BCL2 expression, promoting apoptosis and reducing invasion and migration abilities [12]. This compound can also modulate similar effects through the PI3K/Akt/mTOR pathway, the NF-κB system, and the Wnt/β-catenin pathway [13]. Meanwhile, curcumin has been shown to induce apoptosis in cancer cells by inhibiting glycolysis and the PI3K/Akt signaling pathway, as well as significantly reducing tumor volume and weight in prostate cancer models in vivo studies, indicating its potential as an effective chemo-preventive agent [14].

**Fig. 1:**
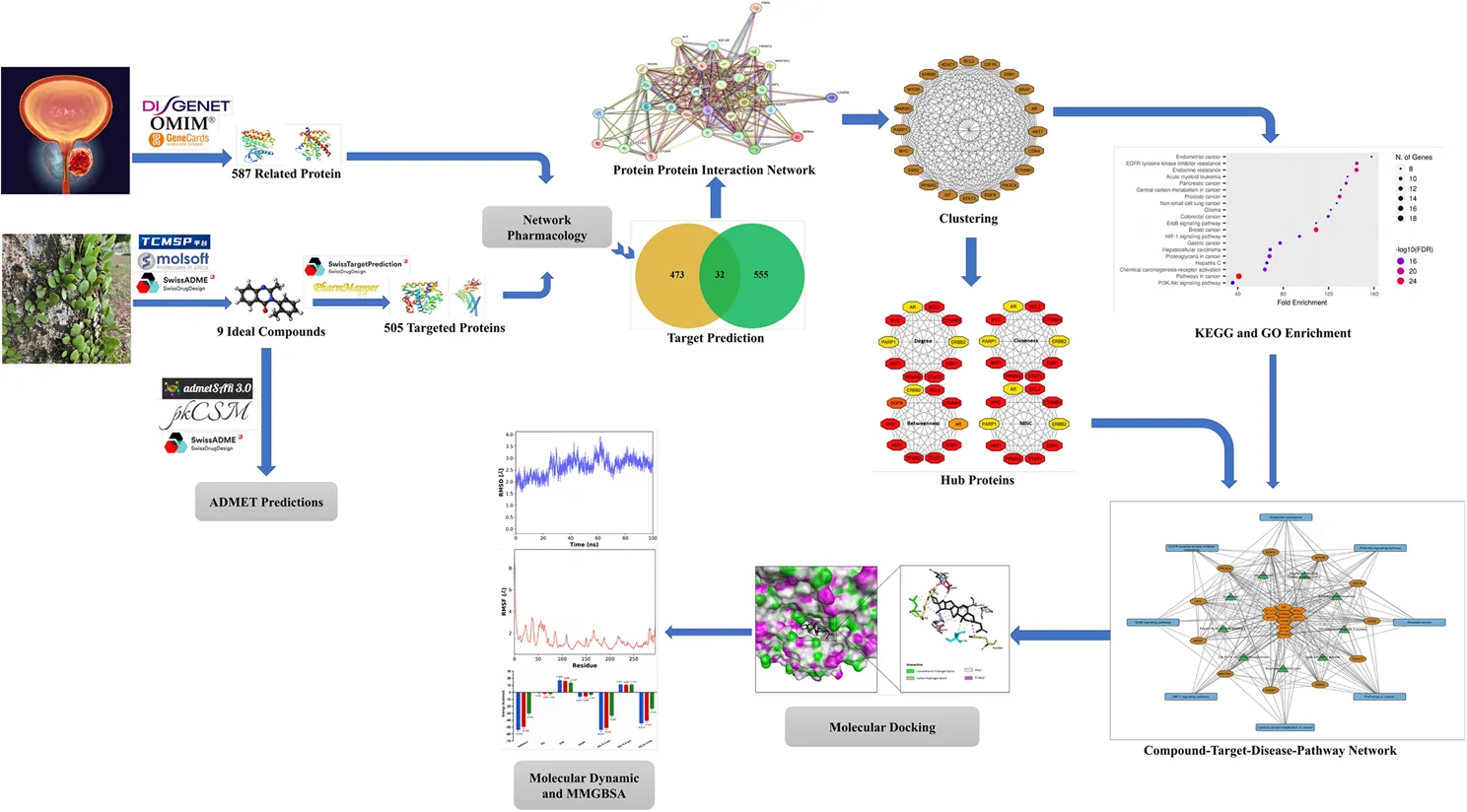
Workflow of an integrated network nharmacology and molecular simulation approach to investigate the potential mechanism of *Pyrrosia piloselloides* against prostate cancer used in this study

Despite advances in prostate cancer research, the pharmacological effects and molecular mechanisms of compounds from the dragon scale plant in this disease remain poorly characterized. In response to this gap, network pharmacology provides a systematic approach to identify prostate cancer–related targets associated with multiple bioactive compounds, thereby enabling the identification of key proteins involved in the disease. The potential binding of these compounds to the identified key targets can then be further evaluated using molecular docking, which predicts binding affinities and characterizes ligand–protein interaction patterns [15–20]. However, molecular docking provides a static representation of ligand–protein interactions and does not fully capture their dynamic behavior. Molecular dynamics (MD) simulation can therefore be applied following docking to evaluate the stability, conformational flexibility, and persistence of ligand–protein interactions using parameters such as RMSD, RMSF, hydrogen-bond occupancy, and MM/PBSA or MM/GBSA binding free energy [18, 21]. Taken together, integrating network pharmacology, molecular docking, and MD simulation thus provides a comprehensive computational framework for characterizing compound–target interactions and prioritizing compounds for subsequent experimental investigation [22–26]. Thus, the study aims to analyse the effects and underlying mechanisms of bioactive compounds from the *P. piloselloides* on prostate cancer using a combination of network pharmacology, molecular docking, and molecular dynamics. The study workflow is presented in Fig. 1.

## Materials and methods

### Retrieval and Screening of *P. piloselloides* compounds 

All dragon scale fern compounds were collected from the relevant literature, in which GC–MS analysis of the aqueous and methanolic leaf extracts of *Pyrrosia piloselloides* identified 59 unique bioactive compounds [9]. These compounds were subsequently screened using five criteria: molecular weight (MW) < 500 Da, hydrogen bond acceptors (HBA) < 10, hydrogen bond donors (HBD) < 5, oral bioavailability (OB) ≥ 30%, and drug-likeness (DL) ≥ 0.18. The OB and DL thresholds were selected because they are widely adopted in network pharmacology studies to prioritize compounds with favorable predicted pharmacokinetic properties while minimizing the inclusion of compounds with poor oral absorption or low drug-likeness. Specifically, an OB threshold of 30% is commonly used to identify compounds with acceptable predicted oral absorption, whereas a DL threshold of 0.18 corresponds to the average drug-likeness index of approved drugs in the DrugBank database. Screening was performed using SwissADME, Molsoft, and TCMSP to evaluate the compounds on five predefined criteria. Only compounds that met all five criteria were retained for subsequent analyses [22, 23].

### Analysis of *Pyrrosia piloselloides* target proteins associated with prostate cancer

Potential protein targets of the bioactive compounds identified from *Pyrrosia piloselloides* were predicted using two complementary target prediction platforms, SwissTargetPrediction and PharmMapper, to improve target coverage and prediction reliability. For SwissTargetPrediction, the canonical SMILES of each compound was used as the query input, and target prediction was performed using the default settings with the species restricted to *Homo sapiens*. Only predicted targets with a probability score > 0 were retained for subsequent analysis. For PharmMapper, three-dimensional conformers were generated for each compound by enabling the conformer generation option with a maximum of 300 conformations. Reverse pharmacophore mapping was performed against the Human Protein Targets Only database (v2010, 2,241 targets), and up to 300 matched targets were retained for each compound. To improve prediction reliability, only targets with a normalized fit score (Z-score) > 1.5 were included in the final dataset. The predicted targets obtained from SwissTargetPrediction and PharmMapper were subsequently merged. Because the scoring systems of SwissTargetPrediction and PharmMapper are based on different prediction algorithms, confidence scores were not directly compared. Instead, predefined filtering criteria were applied independently to each platform before integration.

Prostate cancer-associated proteins were retrieved from the GeneCards, OMIM, and DisGeNET databases using the keyword “Prostate Cancer”. To prioritize genes with strong evidence of association, only GeneCards entries with a relevance score ≥ 30 were included, consistent with previous network pharmacology studies. This threshold was selected to enrich the dataset with genes supported by stronger biological and biomedical evidence while minimizing the inclusion of weakly associated targets. Protein lists obtained from the three databases were subsequently merged, and duplicate entries were removed to establish a comprehensive set of prostate cancer-related targets. Finally, the intersection between the predicted targets of dragon scale fern compounds and prostate cancer-associated proteins was identified using jvenn. The overlapping proteins were defined as candidate therapeutic targets for the subsequent network pharmacology and molecular docking analyses [24, 27–29].

### Construction of the Protein-Protein Interaction (PPI) network

The protein–protein interaction (PPI) network of the overlapping targets was constructed using the STRING database (version 12.0) by restricting the analysis to *Homo sapiens*. A minimum required interaction score of 0.4 (medium confidence) was applied to retain experimentally supported and computationally predicted protein interactions while minimizing weak associations. The resulting interaction network was imported into Cytoscape (version 3.10.1) for visualization and topological analysis, and isolated nodes without interactions were excluded from the primary network [27].

### Clustering analysis and hub gene identification

Clustering analysis and hub gene identification were performed using MCODE and CytoHubba plugins in Cytoscape. Default settings were applied for clustering, with the highest-scoring cluster highlighted. Hub gene identification was based on four parameters: degree centrality (DC), betweenness centrality (BC), closeness centrality (CC), and Maximum Neighborhood Component (MNC). Top 10 scoring proteins from each parameter were further examined and intersected using a Venn diagram to identify key/core proteins associated prostate cancer targeted by the *P. piloselloides *compouds [23, 27].

### Gene ontology enrichment and KEGG pathway analysis

Gene Ontology (GO) and Kyoto Encyclopedia of Genes and Genomes (KEGG) pathway enrichment analyses were performed using ShinyGO (version 0.80), with *Homo sapiens* selected as the reference organism. The analyses were conducted on the top protein cluster identified from the clustering analysis. GO enrichment included biological process (BP), molecular function (MF), and cellular component (CC) categories, while KEGG analysis was used to identify significantly enrichment pathway associated with prostate cancer. Statistical significance was determined using a false discovery rate (FDR) < 0.05, and the top 20 enriched GO terms and KEGG pathways were visualized as dot plots [30].

### Molecular docking analysis

Molecular docking was performed using AutoDock Vina and MGL Tools 1.5.6 package to evaluate the binding affinity of *P. piloselloides* -derived compounds toward key targets associated with prostate cancer. The selected protein targets included ESR1 (PDB ID: 2IOK), MYC (PDB ID: 1NKP), CTNNB1 (PDB ID: 7AFW), PPARG (PDB ID: 2PRG), AKT1 (PDB ID: 4GV1), STAT3 (PDB ID: 6NJS), BCL-2 (PDB ID: 4IEH), AR (PDB ID: 2AX9), and ERBB2 (PDB ID: 3PP0), all of which were retrieved from the RCSB Protein Data Bank.

Protein preparation involved the removal of co-crystallized ligands, water molecules, and other non-essential heteroatoms, followed by the addition of polar hydrogen atoms and partial charges. Ligand structures were geometry-optimized using Avogadro and subsequently processed in AutoDock by assigning Gasteiger partial charges and polar hydrogen atoms. Both protein and ligand structures were then converted into the .pdbqt format required for molecular docking simulations.

Docking grid boxes were centered on the native ligand-binding sites based on the coordinates of the co-crystallized ligands (Table S1). To validate the docking protocol, each co-crystallized ligand was re-docked into its corresponding binding pocket. The docking protocol was considered valid when the root mean square deviation (RMSD) between the docked and crystallographic ligand poses was less than 2.0 Å. After successful validation, the prepared protein structures were docked with the test compounds using the same docking parameters. The resulting protein–ligand binding poses and molecular interactions were subsequently analyzed and visualized using BIOVIA Discovery Studio Visualizer 2021 [30, 31].

### Dynamic Simulation and MM-GBSA Calculation

Molecular dynamics (MD) simulations were performed using OpenMM with the AMBER force fields implemented on the Google Colab platform to investigate the stability and dynamic behavior of the protein–ligand complexes. Protein topologies were generated using the ff19SB force field, while ligand parameters were assigned using the General AMBER Force Field 2 (GAFF2). Each system was solvated in a cubic simulation box with a 12 Å buffer using the TIP3P explicit water model and neutralized with 0.15 M NaCl. Prior to the production run, energy minimization was followed by equilibration under the isothermal–isobaric (NPT) ensemble at 310 K and 1 atm for 0.3 ns. Subsequently, production MD simulations were carried out for 100 ns with a 2 fs integration time step, and trajectory coordinates were saved every 10 ps for subsequent analyses. Binding free energies were estimated using the Molecular Mechanics/Generalized Born Surface Area (MM/GBSA) method with the Generalized Born model (igb = 2). The binding free energy was calculated according to the following equations [19, 30, 32]:

$$\:{\Delta\:}{G}_{\mathrm{bind}}={G}_{\mathrm{complex}}-({G}_{\mathrm{receptor}}+{G}_{\mathrm{ligand}})$$

### Prediction of the Absorption, Distribution, Metabolism, Excretion, and Toxicity Profiles of *P. piloselloides* Compounds

The absorption, distribution, metabolism, excretion, and toxicity (ADMET) profiles of the *P. piloselloides* compounds were predicted using the pkCSM (https://biosig.lab.uq.edu.au/pkcsm/), SwissADME (http://www.swissadme.ch/index.php), and admetSAR (https://lmmd.ecust.edu.cn/admetsar3/predict.php) tools to evaluate the safety of these compounds [30].

## Result

### Screening of *P. piloselloides* compounds 

A total of 59 *P. piloselloides* compounds were retrieved from GC-MS profiling conducted by Sul'ain et al [9] and further screened using tools such as SwissADME, Molsoft, and TCMSP. After evaluating, eight compounds met all five screening parameters, including 6-Hydroxymethaqualone, 2,6,10,14,18,22-Tetracosahexaene, Ergost-8(14)-en-3beta-ol, Stigmasterol, Chondrillast-7-enol, Stigmastan-3,5,22-trien, 7-Dehydrodiosgenin 3-acetate, and Beta-Sitosterol acetate (Table 1). These compounds were selected for further analysis in subsequent stages.

**Table 1:** *P. piloselloides* compounds compounds that passed screening and were selected as test compounds

Name Of Compound	Molecular Formula	SwissAdme				TCMSP	Molsoft
		Bioavailability (≥ 30%)	Molecular Weight (≤ 500 g/mol)	Number of HBA (≤ 10)	Number of HBD (≤ 5)	Druglikeness (≥ 0.18)	Druglikeness (≥ 0.18)
6-Hydroxymethaqualone	C16H14N2O2	0.55	266.29 g/mol	3	1	**-**	0.6
2,6,10,14,18,22-Tetracosahexaene	C30H50	0.55	410.72 g/mol	0	0	0.42	-0.9
Ergost-8(14)-en-3-ol, (3.beta.)-	C28H48O	0.55	400.68 g/mol	1	1	-	0.46
Stigmasterol	C29H48O	0.55	412.69 g/mol	1	1	0.76	0.62
Stigmast-7-en-3-ol, (3.beta.,5.alpha.,24 S)-	C29H50O	0.55	414.71 g/mol	1	1	0.75	0.18
Stigmastan-3,5,22-trien	C29H46	0.55	394.68 g/mol	0	0	**-**	0.35
7-Dehydrodiosgenin 3-acetate	C29H42O4	0.55	454.64 g/mol	4	0	**-**	0.32
.beta.-Sitosterol acetate	C31H52O2	0.55	456.74 g/mol	2	0	0.85	1.11

### Potential targets of *P. piloselloides* compounds in prostate cancer

**Fig. 2:**
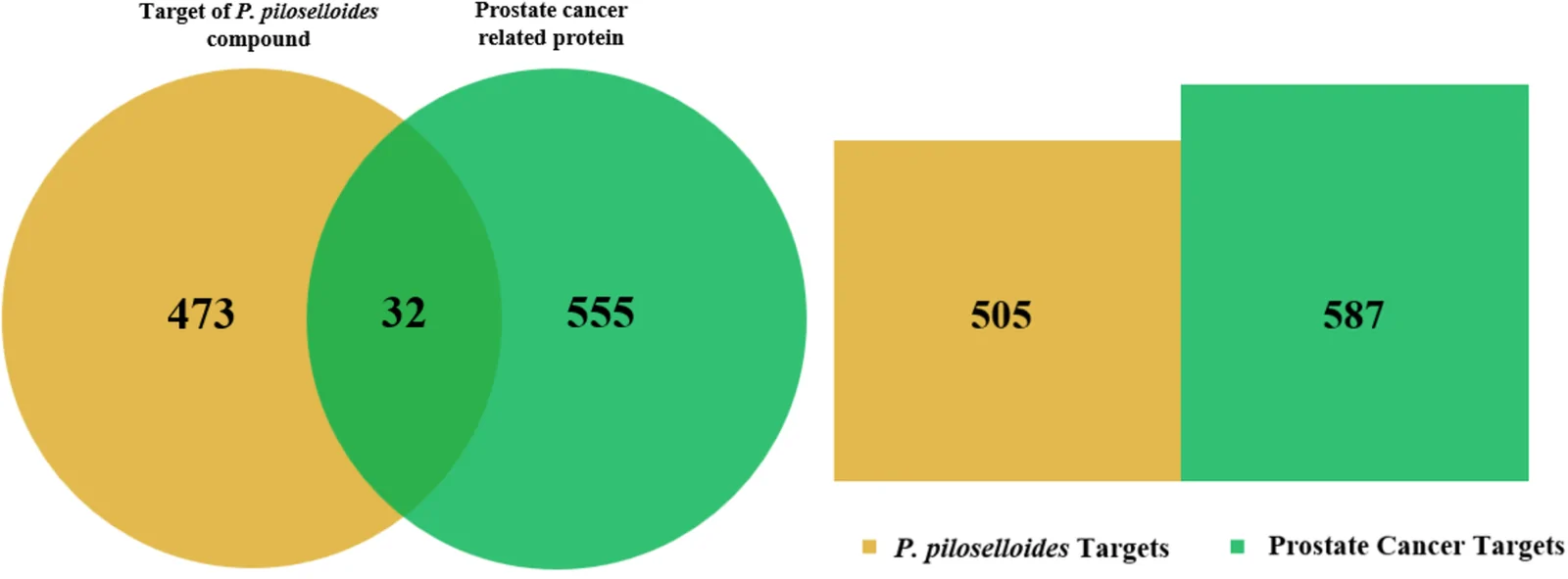
Venn diagram analysis intersection of prostate cancer related target protein with *P. piloselloides* compound target

The potential protein targets of *P. piloselloides* compounds were predicted using PharmMapper and SwissTargetPrediction. After merging the retrieved targets and removing duplicates, a total of 505 unique proteins were identified as potential targets of the compounds. In parallel, prostate cancer–related proteins were collected from the GeneCards, OMIM, and DisGeNET databases. Following removal of redundant entries, 587 unique disease-associated proteins were obtained. The two protein sets were subsequently compared using a Venn diagram, which identified 32 overlapping proteins. These shared proteins were considered potential targets linking *P. piloselloides* compounds to prostate cancer and were subsequently subjected to further network and molecular analyses. (Fig. 2).

### Network PPI construction, clustering, and hub protein identification

**Fig. 3:**
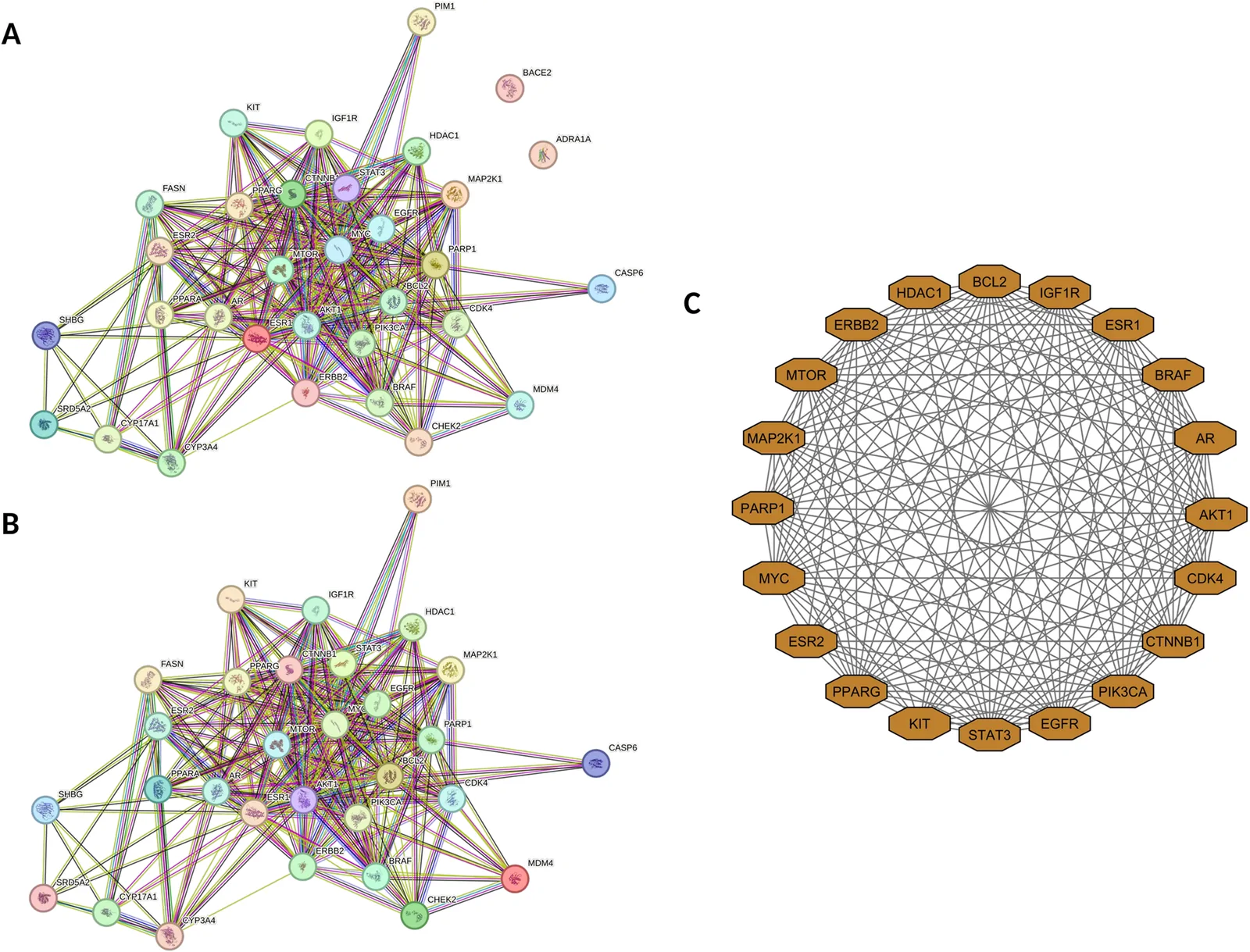
Protein–protein interaction network of prostate cancer related protein targeted by *P. piloselloides* compounds. **A** The PPI network of 32 protein related prostate cancer targeted by the plants compouds according to STRING. **B** The PPI network of 30 protein target of the compound target in prostate cancer after clearing non interaction protein with main network. **C** Visualization of major cluster of protein–protein interaction network of the compounds targets in prostate cancer

The 32 prostate cancer proteins targeted by the *P. piloselloides* compounds were further analysed to determine protein-protein network interactions and topological properties. Protein-protein interactions were constructed using STRING (an online tool) and visualized using the Cytoscape software package. Higher node scores indicated a more critical role in the PPI network (Table 2). The PPI results (Fig. 3a) revealed that some proteins did not interact with others and were removed from the main network using Cytoscape. The final network consisted of 30 protein nodes and 250 edges (Fig. 3b). Network clustering was performed using the MCODE plugin in Cytoscape, generating three distinct clusters. The highest-scoring cluster, with a score of 18.105, was identified as the major cluster, containing 20 protein nodes and 172 edges (Fig. 3c).

**Table 2:** Topological of protein related prostate cancer targeted by selected *P. piloselloides* compounds

No	Name	Closeness	Degree	Betweeneess	MNC
1	PIM1	0.492	3	0.0	23.333
2	CYP3A4	0.63	12	0.012	17.75
3	PPARA	0.644	13	0.003	20.692
4	CYP17A1	0.569	8	0.003	16.125
5	ERBB2	0.806	22	0.015	19.273
6	PIK3CA	0.763	20	0.007	20.05
7	MYC	0.853	24	0.036	18.333
8	BRAF	0.707	17	0.002	21.059
9	CDK4	0.725	18	0.004	20.222
10	SRD5A2	0.527	6	2.463	15.333
11	HDAC1	0.659	14	7.606	22.143
12	PARP1	0.805	22	0.027	18.773
13	EGFR	0.829	23	0.038	18.348
14	BCL2	0.829	23	0.031	18.609
15	FASN	0.644	13	0.006	20.615
16	IGF1R	0.707	17	0.002	21.353
17	AKT1	0.906	26	0.062	17.615
18	AR	0.829	23	0.047	18.174
19	ESR1	0.906	26	0.067	17.577
20	MAP2K1	0.69	16	0.002	21.312
21	KIT	0.659	14	1.535	22.214
22	MTOR	0.829	23	0.017	18.869
23	CHEK2	0.644	13	0.001	21.0
24	MDM4	0.591	11	5.554	21.0
25	SHBG	0.537	7	0.001	16.285
26	STAT3	0.829	23	0.031	18.695
27	CTNNB1	0.784	21	0.008	19.809
28	CASP6	0.5	3	0.0	23.667
29	ESR2	0.69	16	0.023	19.062
30	PPARG	0.829	23	0.026	18.826

**Fig. 4:**
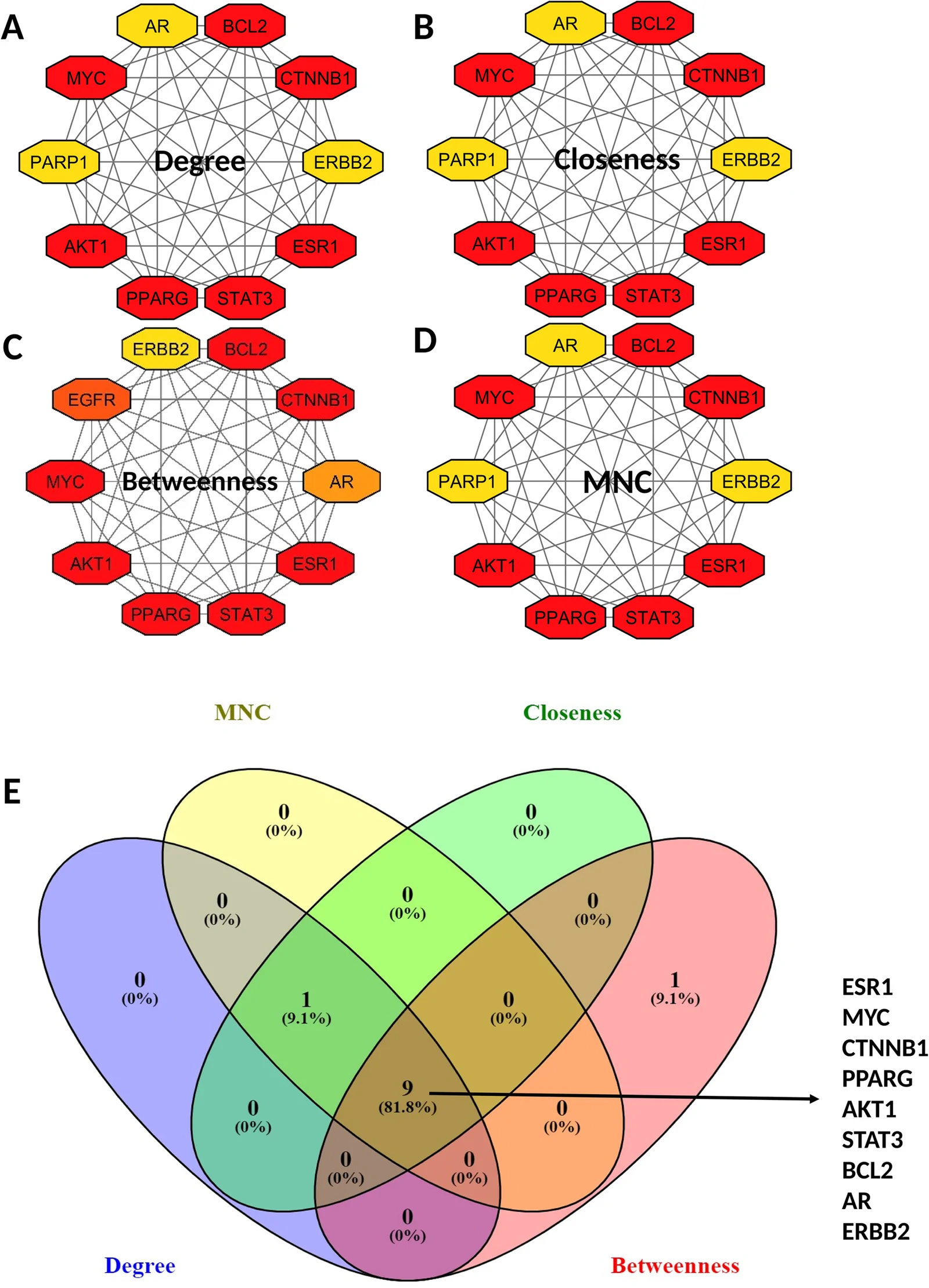
Top 10 Hub gene in diferent centrality. **A** Degree **B** Closeness **C** Betweennesse **D** Maximum Neighborhood Component **E** Venn diagram of top 10 Hub gene that figurize nine obtained key proteins including ESR1, MYC, CTNNB1, PPARG, AKT1, STAT3, BCL2, AR, and ERBB2

Using the CytoHubba plugin, proteins within the primary cluster were ranked according to four centralities: degree (DC), betweenness (BC), closeness centrality (CC), and maximum neighbourhood component (MNC). Based on the same plugins and centrality parameter, key proteins were screened to identify the most potent proteins for prostate cancer targeted by the *P. piloselloides* compounds. The top ten proteins for each centrality parameter (Hub genes) were determined and compared using a Venn diagram (Fig. 4a-d). This analysis revealed nine key interacting proteins: ESR1, MYC, CTNNB1, PPARG, AKT1, STAT3, BCL2, AR, and ERBB2, which were suggested as key proteins (Fig. 4e).

### GO enrichment and KEGG pathway analysis

**Fig. 5:**
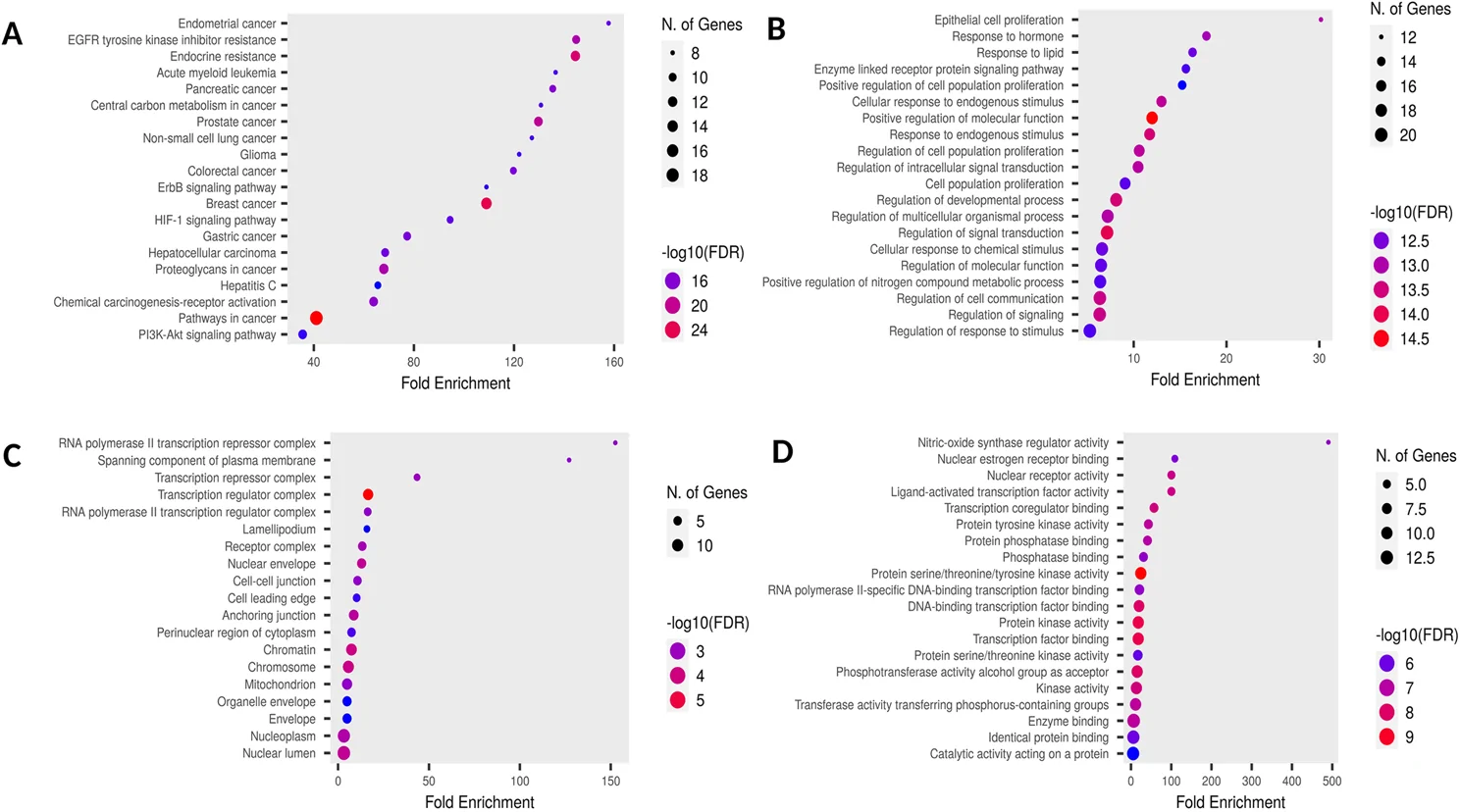
Gene Ontology (GO) and KEGG pathway enrichment analysis of the 20 proteins comprising the major cluster. **A** Top 20 enriched Molecular Function **B** Top 20 enriched Cellular Component. **C** Top 20 enriched Biological Process. **D** Top 20 enriched KEGG pathways

Gene Ontology (GO) enrichment was performed to assess molecular functions, cellular components, and biological processes associated with prostate cancer targeted by *P. piloselloides* compounds. The aim was to provide a comprehensive understanding of molecular, cellular, and biological changes in prostate cancer upon exposure to compounds. A total of 235 molecular function terms were identified, among which the top 20 enriched terms are presented in Fig. 5A. *Nitric oxide synthase regulator activity* exhibited the highest enrichment, suggesting a potential involvement of the nitric oxide (NO)/nitric oxide synthase (NOS) axis in the biological activity of the investigated compounds. This signaling axis has been implicated in several aspects of prostate cancer biology. Other prostate cancer-relevant molecular functions were also among the top enriched terms, including nuclear estrogen receptor binding and nuclear receptor activity, which ranked second and third, respectively.

For the CC analysis, 142 cellular component terms were identified based on the input proteins. The top 20 enriched cellular components are shown in Fig. 5B, with the RNA polymerase II transcription repressor complex among the most highly enriched terms. Nucleoplasm was also identified as a significantly enriched cellular component based on its false discovery rate (FDR), suggesting that the predicted targets may be associated with nuclear regulatory processes.

In the BP analysis, a total of 1,000 biological process terms were identified. The top 20 enriched biological processes are presented in Fig. 5C, with epithelial cell proliferation showing the highest enrichment. Several other processes with established relevance to prostate cancer biology were also represented among the top 20 terms, including response to hormone, regulation of signaling, and regulation of cell population proliferation. These findings suggest that the predicted targets of the *P. piloselloides* compounds are involved in biological processes related to cellular proliferation and hormonal and signaling regulation, which are important features of prostate cancer progression.

**Fig. 6:**
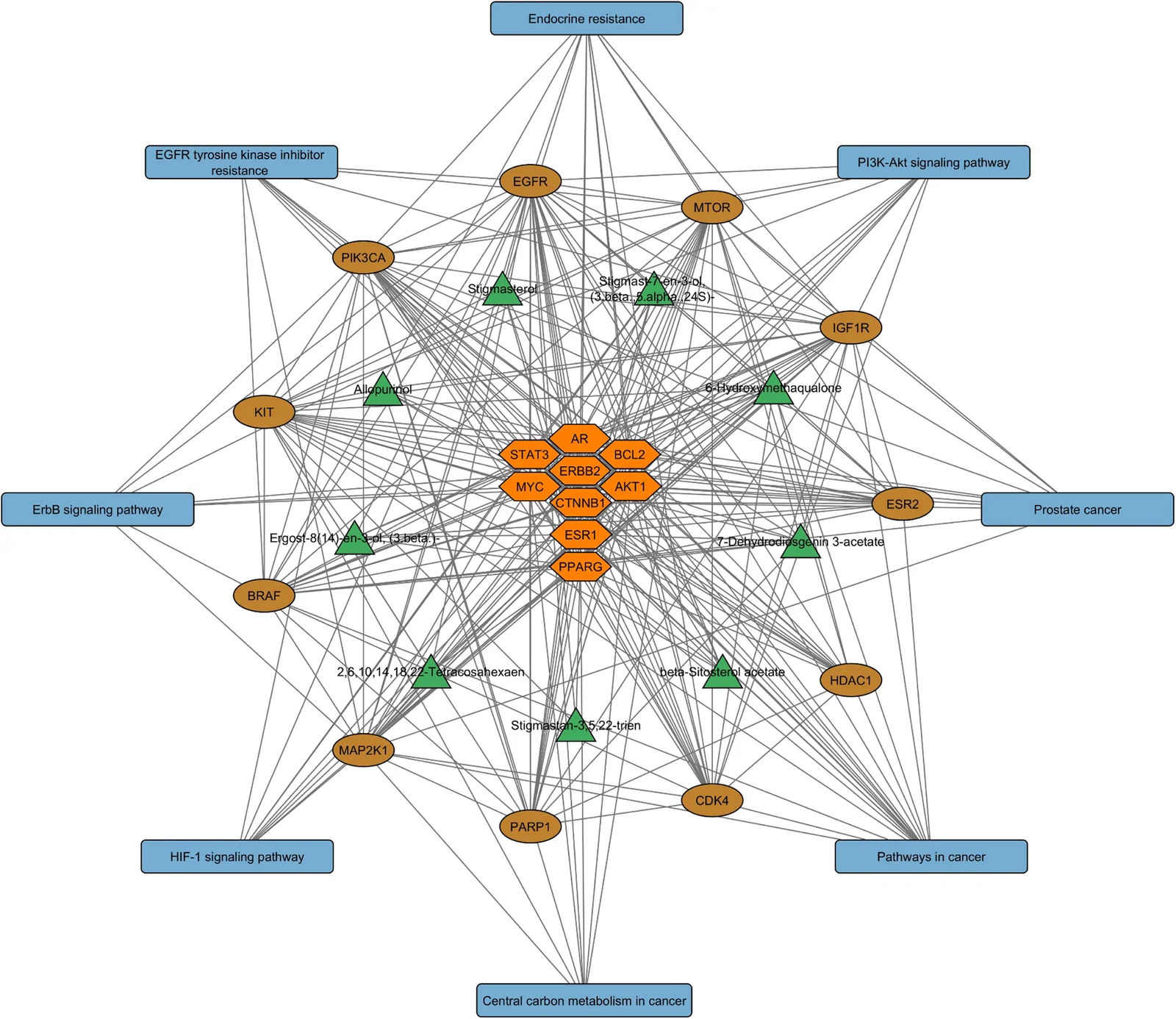
Compound–target–disease-pathway network with 9 potential key protein

**Fig. 7:**
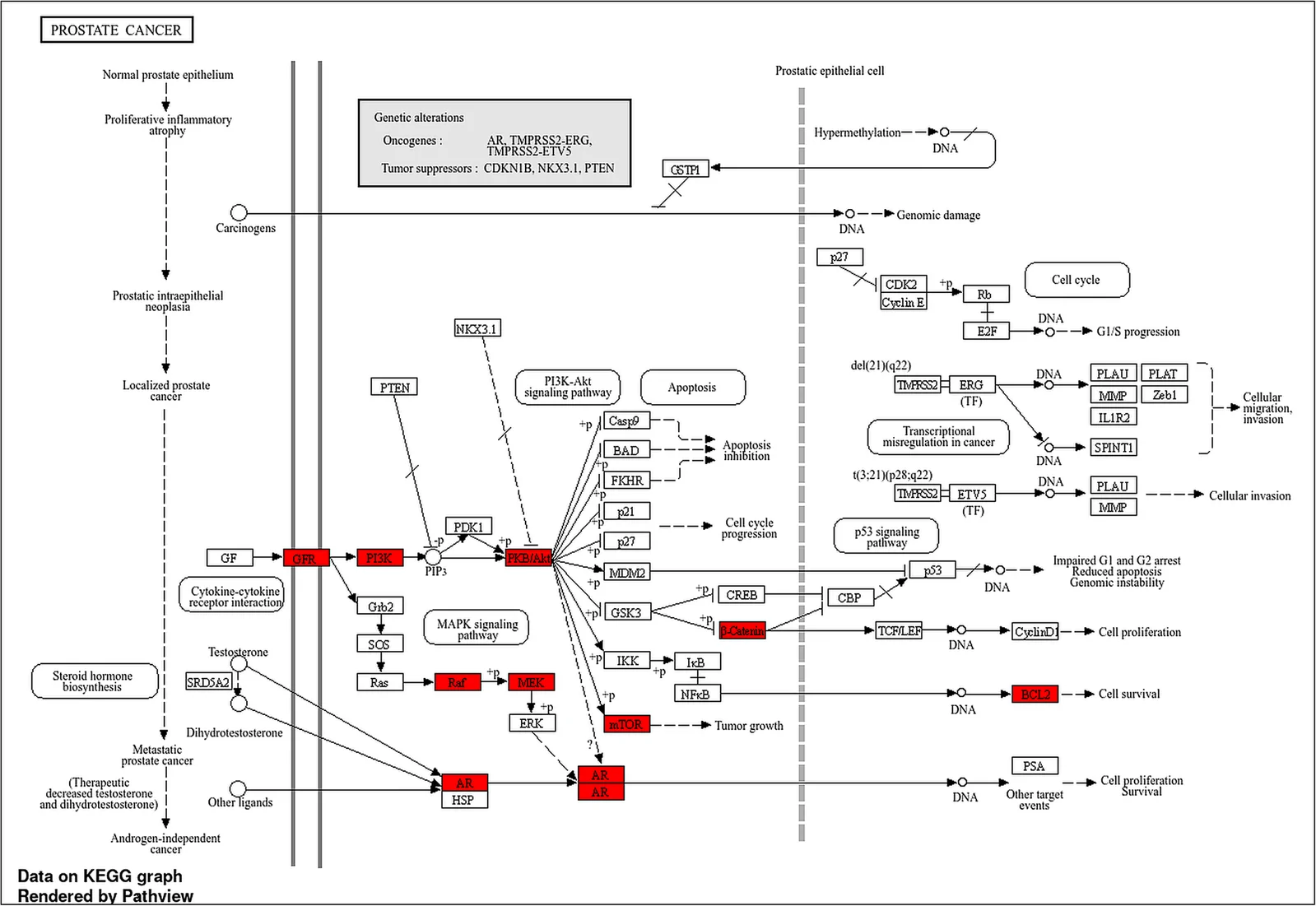
Prostate cancer pathway as one of KEGG pathway enriched

KEGG pathway enrichment analysis was subsequently performed to identify signaling pathways associated with the prostate cancer-related proteins targeted by the investigated compounds. A total of 161 pathways were identified based on the 20 input proteins derived from the primary cluster. The top 20 enriched pathways were ranked according to fold enrichment, with statistical significance assessed using the false discovery rate (FDR) depicted in Figs. 5D, 6 and 7. Notably, eight pathways among the enriched pathways have been reported in the literature to have strong relevance to prostate cancer biology. These included endocrine resistance, PI3K–Akt signaling pathway, prostate cancer, pathways in cancer, central carbon metabolism in cancer, HIF-1 signaling pathway, ErbB signaling pathway, and EGFR tyrosine kinase inhibitor resistance [33–38 ]. These eight pathways were then constructed together with the previously obtained data, forming a Compound-Target-Disease-Pathway Network to create the final visualization in the form of network pharmacology (Fig. 6). 

### Molecular docking

Eight compounds were docked with key proteins, including ESR1, MYC, CTNNB1, PPARG, AKT1, STAT3, BCL2, AR, and ERBB2, to verify promising binding score that hypothesized generate therapeutical potential. These compounds included 6-Hydroxymethaqualone; 2,6,10,14,18,22-Tetracosahexaene; Ergost-8(14)-en-3beta-ol; Stigmasterol; Chondrillast-7-enol; Stigmastan-3,5,22-trien; 7-Dehydrodiosgenin 3-acetate; and Beta-Sitosterol acetate. Validation was carried out using molecular docking, revealing that all compounds interacted with the target proteins.

**Fig. 8:**
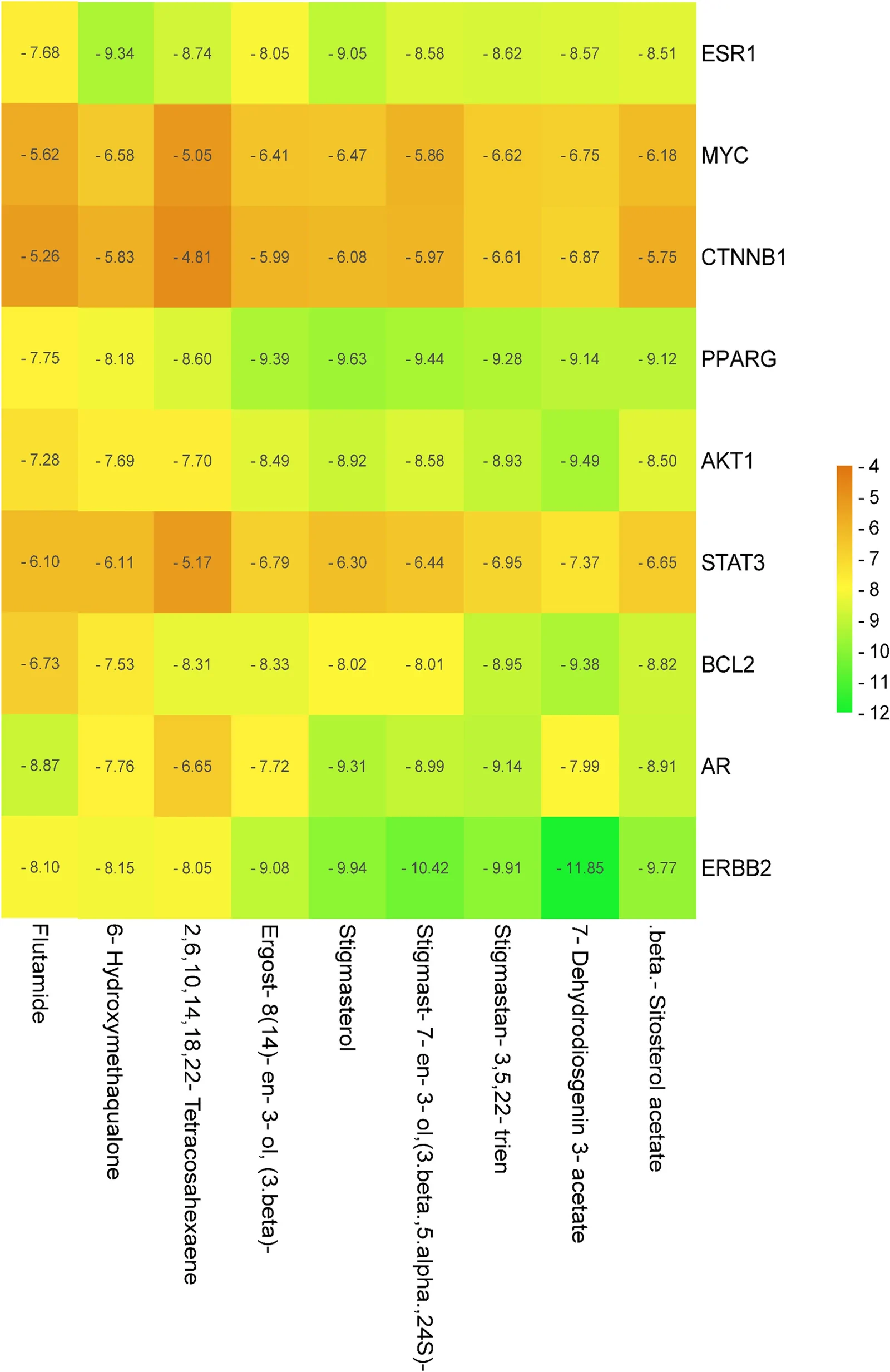
Heat map results of molecular docking of eight *P. piloselloides* compounds against core proteins of prostate cancer

Molecular docking analysis revealed variable predicted binding affinities of the evaluated *Pyrrosia piloselloides* compounds across the nine prostate cancer-related protein targets (Fig. 8). Together that, several compounds exhibited more negative docking scores than the reference compounds for one or more targets, indicating relatively favorable predicted ligand–target interactions. Among the evaluated compounds, 7-dehydrodiosgenin 3-acetate showed the most negative docking scores for six targets, namely MYC (ΔG = − 6.75 kcal/mol), CTNNB1 (ΔG = − 6.87 kcal/mol), AKT1 (ΔG = − 9.49 kcal/mol), STAT3 (ΔG = − 7.37 kcal/mol), BCL2 (ΔG = − 9.38 kcal/mol), and ERBB2 (ΔG = − 11.85 kcal/mol). Stigmasterol showed the most negative scores for PPARG (ΔG = − 9.63 kcal/mol) and AR (ΔG = − 9.31 kcal/mol), whereas 6-hydroxymethaqualone exhibited the most negative score for ESR1 (ΔG = − 9.34 kcal/mol). Notably, the most favorable docking score was observed for the 7-dehydrodiosgenin 3-acetate–ERBB2 complex (− 11.85 kcal/mol), followed by stigmasterol–ERBB2 (− 10.42 kcal/mol) and stigmasterol–PPARG (− 9.63 kcal/mol).

**Fig. 9:**
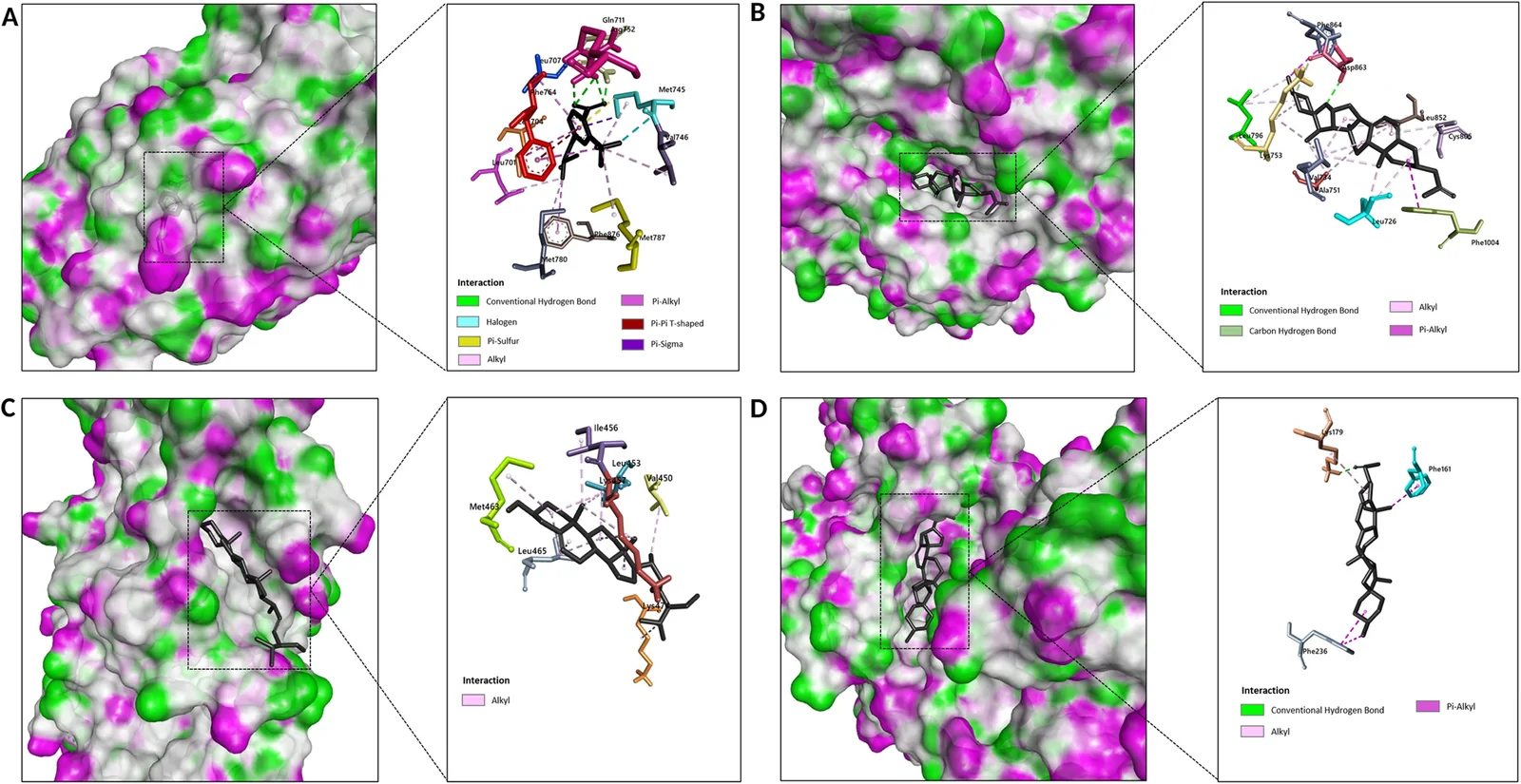
Visualization 3D of molecular docking results of best protein–ligand (*P. piloselloides* compounds and positive control) complexes. **A** AR – Flutamide (control). **B** ERBB2–6-Hydroxymethaqualone. **C** PPARG – Stigmasterol. **D** AKT1–7-Dehydrodiosgenin 3-acetate

**Fig. 10:**
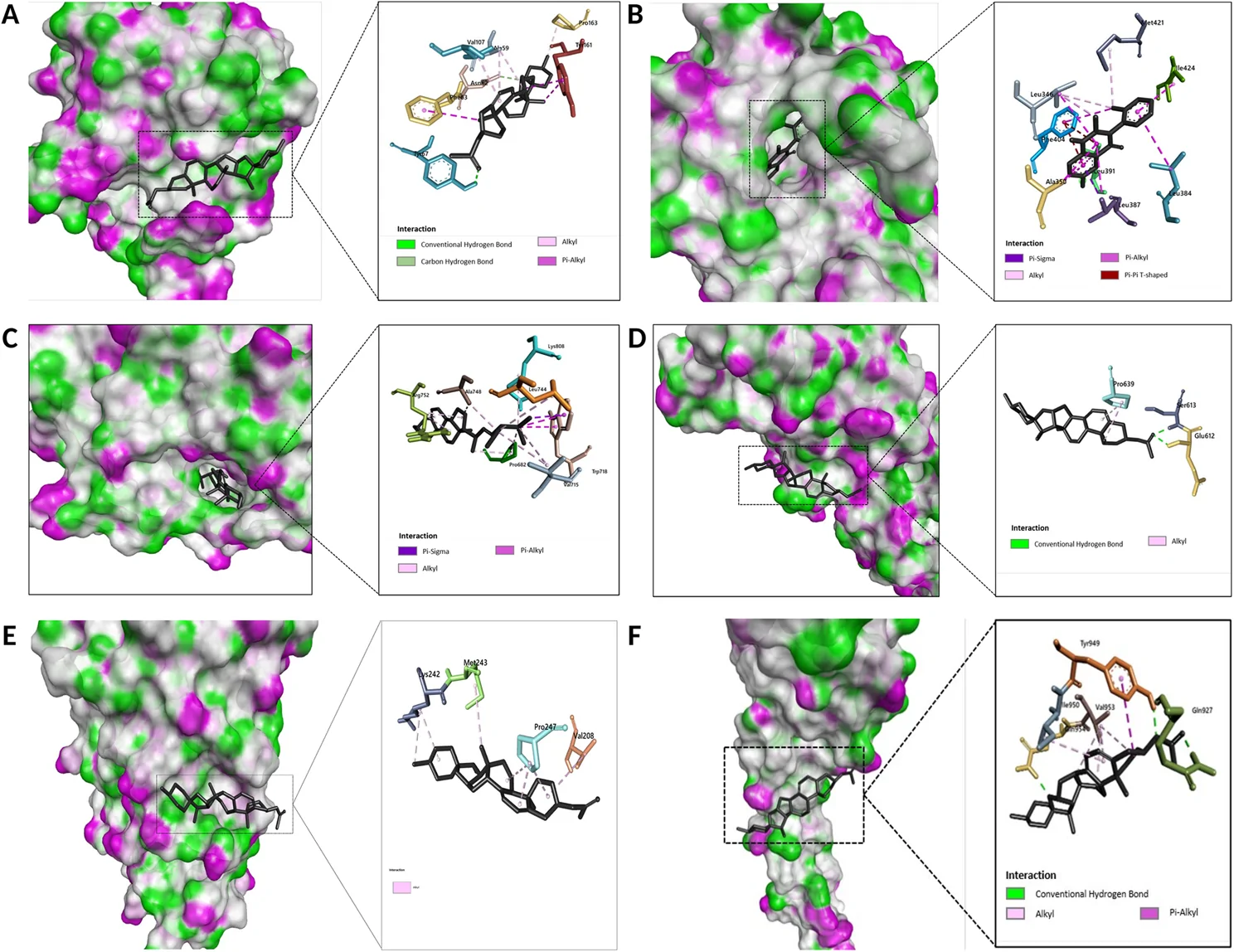
Visualization 3D of molecular docking results of best protein–ligand (*P. piloselloides* compounds and positive control) complexes. **A** BCL2–7-Dehydrodiosgenin 3-acetate. **B** ESR1–7-Dehydrodiosgenin 3-acetate. **C** AR – Stigmasterol. **D** STAT3–7-Dehydrodiosgenin 3-acetate. **E** CTNNB1–7-Dehydrodiosgenin 3-acetate. **F** MYC − 7-Dehydrodiosgenin 3-acetate

The docked compounds were consistently positioned within the respective active-site regions of their corresponding protein targets, supporting the predicted target–ligand associations and indicating that these interactions may potentially influence target protein activity. Ligand binding involved a range of non-covalent interactions, including hydrogen bonds, van der Waals interactions, π–π T-shaped and π–sigma interactions, carbon–hydrogen bonds, π–sulfur/sulfur-X interactions, alkyl and π-alkyl interactions, and halogen bonding (Figs. 9a–d and 10a–e, and Table 3). 

**Table 3:** Interaction residue key protein target with selected P. piloselloides compound and positive control

Complex docked	Hydrogen bond	Others bond	Shorter distance interaction (Å)
AR - Flutamide	GLN 711 ARG 752	ASN 705^a^, THR 877^a^, MET 742^a^, MET 749^a^, LEU 873^a^, GLY 708^a^, LEU 701^b^, MET 780^b^, VAL 746^b^, MET 787^b^, MET 745^b^, PHE 764^c^, MET 745^d^, PHE 764^e^, LEU 707^e^, LEU 704^e^, PHE 876^e^, MET 745^g^, MET 745^h^	1,90
ESR1 – 6-Hydroxy methaqualone		LEU 349^a^, LEU 525^a^, GLY 521^a^, MET 388^a^, LEU 428^a^, ARG 394^a^, GLU 353^a^, LEU 346^b^, MET 421^b,^ PHE 404^c,^ LEU 387^d^, LEU 387^e^, ALA 350^e^, PHE 404^e^, LEU 391^e^, LEU 384^e^, LEU 346^e^, ILE 424^e^	3,69
MYC - 7-Dehydro diosgenin 3-acetate	GLN 927 TYR 949 GLN 954	VAL 953^b^, ILE 950^b^, TYR 949^e^	1,97
CTNNB1 – 7-Dehydro diosgenin 3-acetate		GLN 203^a^, ASN 204^a^, THR 205^a^, SER 246^a^, ASN 206^a^, VAL 248^a^, LYS 242^b^, MET 243^b^, PRO 247^b^, VAL 208^b^	4,16
PPARG - Stigmasterol		GLN 454^a^, TYR 473^a^, ASP 475^a^, PHF 282^a^, GLN 286^a^, SER 464^a^, GLN 283^a^, VAL 450^b^, LYS 474^b^, LYS 457^b^, LEU 453^b^, ILE 456^b^, LEU 465^b^, MET 463^b^	3,89
AKT1 – 7-Dehydro diosgenin 3-acetate	LYS 179	ASP 439^a^, GLU 234^a^, THR 160^a^, GLY 159^a^, ASP 292^a^, GLY 162^a^, ILE 186^a^, GLU 191^a^, GLU 198^a^, LEU 181^a^, LYS 163^a^, VAL 164^a^, GLY 157^a^, LYS 158^a^, GLU 278^a^, PHE 442^a^, LYS 179^b^, PHE 161^e^, PHE 238^e^	2,22
STAT3–7-Dehydrodiosgenin 3-acetate	GLU 612 SER 613	ARG 609^a^, SER 611^a^, SER 614^a^,TRP 623^a^, VAL 637^a^, SER 636^a^, GLU 638^a^, PRO 639^b^	1,88 Å
BCL2 – 7-Dehydro diosgenin 3-acetate	TYR 67	GLN 58^a^, ARG 66^a^, GLY 104^a^, ALA 59^b^, VAL 107^b^, PRO 163^b^, PHE 63^e^, TYR 161^e^, ASP 62^f^	2,56
AR - Stigmasterol		VAL 685^a^, GLU 681^a^, PHE 804^a^, GLY 683^a^, TRP 751^a^, THR 755^a^, ASN 756^a^, VAL 684^a^, MET 745^a^, GLN 711^a^,VAL 715^b^, LEU 744^b^, LYS 808^b^, PRO 682^b^, ALA 748^b^, ARG 752^b^, TRP 718^d^, TRP 718^e^	3,53
ERBB2 – 7-Dehydro diosgenin 3-acetate	ASP 863	LEU 785^a^, SER 783^a^, ARG 849^a^, ASP 808^a^, GLY 804^a^, ILE 752^a^, THR 862^a^, THR 798^a^, CYS 805^b^, LEU 796^b^, LYS 753^b^, ALA 751^b^, VAL 734^b^, LEU 726^b^, LEU 852^b^, PHE 864^e^, PHE 1004^e^, ASP 863^f^	2,54

^a^Van der walls, ^b^Alkyl, ^c^Pi-Pi T-Shaped, ^d^Pi-Sigma, ^e^Pi-Alkyl, ^f^Carbon Hydrogen Bond, ^g^Pi-Sulfur, ^h^Halogen (Fluorine)

### Molecular dynamics and binding free energy

The three protein–ligand complexes exhibited stable structural behavior throughout the 100 ns molecular dynamics simulation. Following an initial equilibration phase, the RMSD profiles remained relatively stable, while the RMSF analysis revealed localized residue fluctuations and the Rg values showed only minor variations, indicating that the overall protein compactness was maintained during the simulation. 

**Fig. 11:**
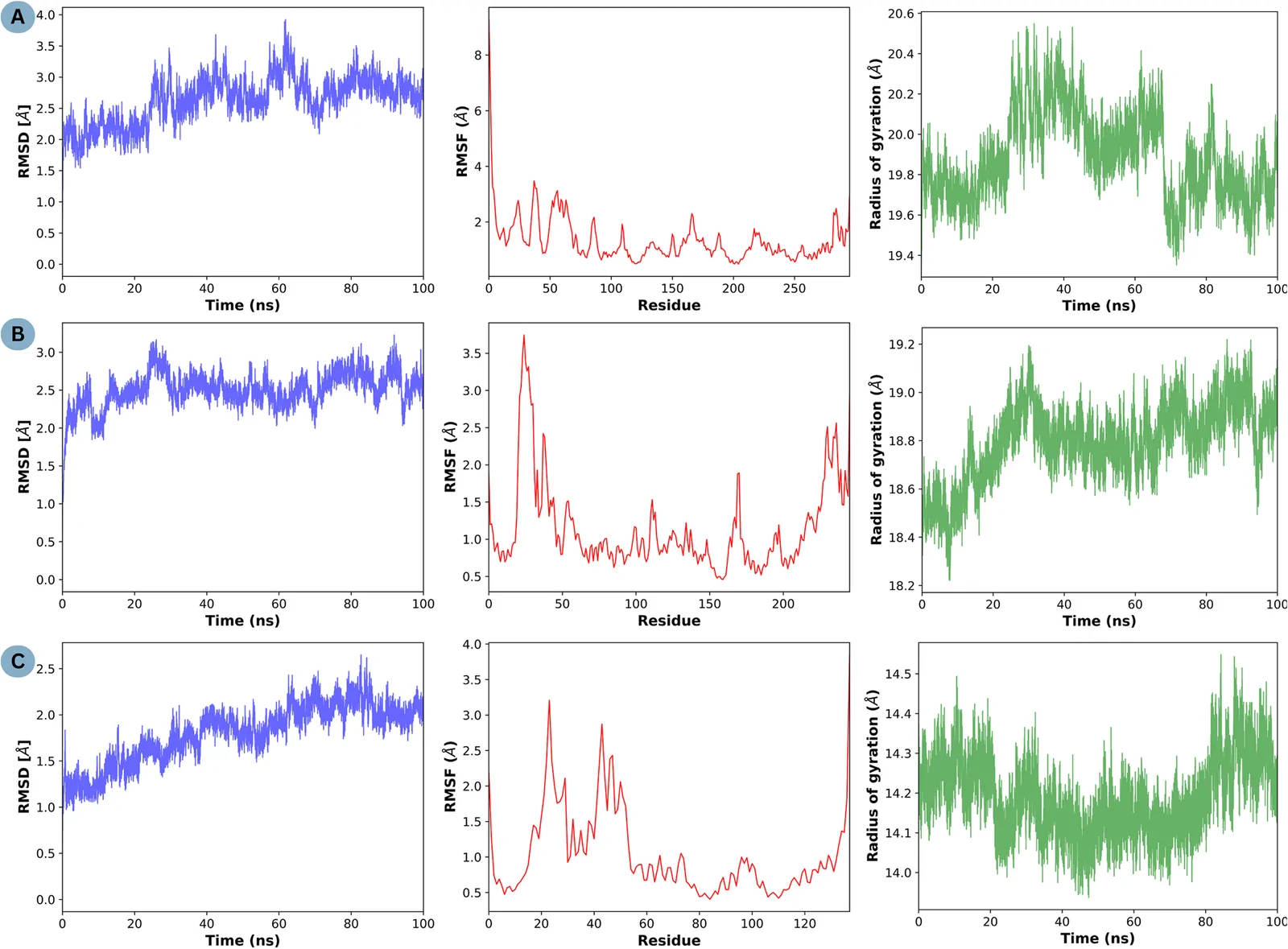
RMSD, RMSF and radius of gyration of selected protein–ligand complexes during molecular dynamic stimulation. **A** ERBB2 / 6-Hydroxymethaqualone. **B** PPARG / Stigmasterol. **C.** BCL2–7-Dehydrodiosgenin 3-acetate

The ERBB2–6-Hydroxymethaqualone complex (Fig. 11A) exhibited an initial RMSD of approximately 1.8 Å, followed by a gradual increase during the first 30 ns before reaching a relatively stable plateau between 2.5 and 3.2 Å for the remainder of the simulation. The corresponding RMSF profile showed that most residues fluctuated below 2.0 Å, while pronounced fluctuations were observed primarily at the N-terminal region, with a maximum value approaching 9 Å. Despite these localized fluctuations, the radius of gyration remained relatively constant within 19.5–20.5 Å, indicating that the elevated residue mobility was restricted to flexible terminal regions and did not induce substantial changes in the overall structural compactness of the protein. The agreement between the stabilized RMSD trajectory and the nearly constant Rg profile suggests that the complex maintained global conformational stability throughout the simulation.

The PPARG–Stigmasterol complex (Fig. 11B) showed a rapid increase in RMSD during the initial equilibration phase, reaching approximately 2.5 Å within the first 20 ns, after which the trajectory fluctuated within a narrow range of 2.3–2.9 Å until the end of the simulation. The RMSF analysis revealed generally low residue mobility, with localized peaks of approximately 3.5–3.7 Å occurring mainly in terminal and loop regions, whereas the majority of residues exhibited fluctuations below 1.5 Å. These localized increases in flexibility were not accompanied by noticeable changes in the Rg profile, which remained stable between 18.3 and 19.2 Å throughout the simulation. The consistency of the RMSD and Rg trajectories indicates that the observed RMSF peaks reflect intrinsic flexibility of surface-exposed regions rather than global structural instability.

The BCL2–7-Dehydrodiosgenin 3-acetate complex (Fig. 11C) exhibited the lowest RMSD among the three systems, increasing gradually from approximately 1.1 Å to 2.0–2.3 Å, followed by a stable trajectory throughout the remaining simulation. RMSF values were generally below 1.0 Å, with only several loop regions and the C-terminal residues displaying higher fluctuations of approximately 2–3.8 Å. Consistent with the low RMSD values, the Rg profile remained highly stable within a narrow range of 14.0–14.5 Å, indicating that these localized residue fluctuations did not affect the global compactness of the protein. Taken together, the low RMSD, limited RMSF peaks, and stable Rg profile suggest that this complex experienced the smallest structural perturbation during the simulation.

**Fig. 12:**
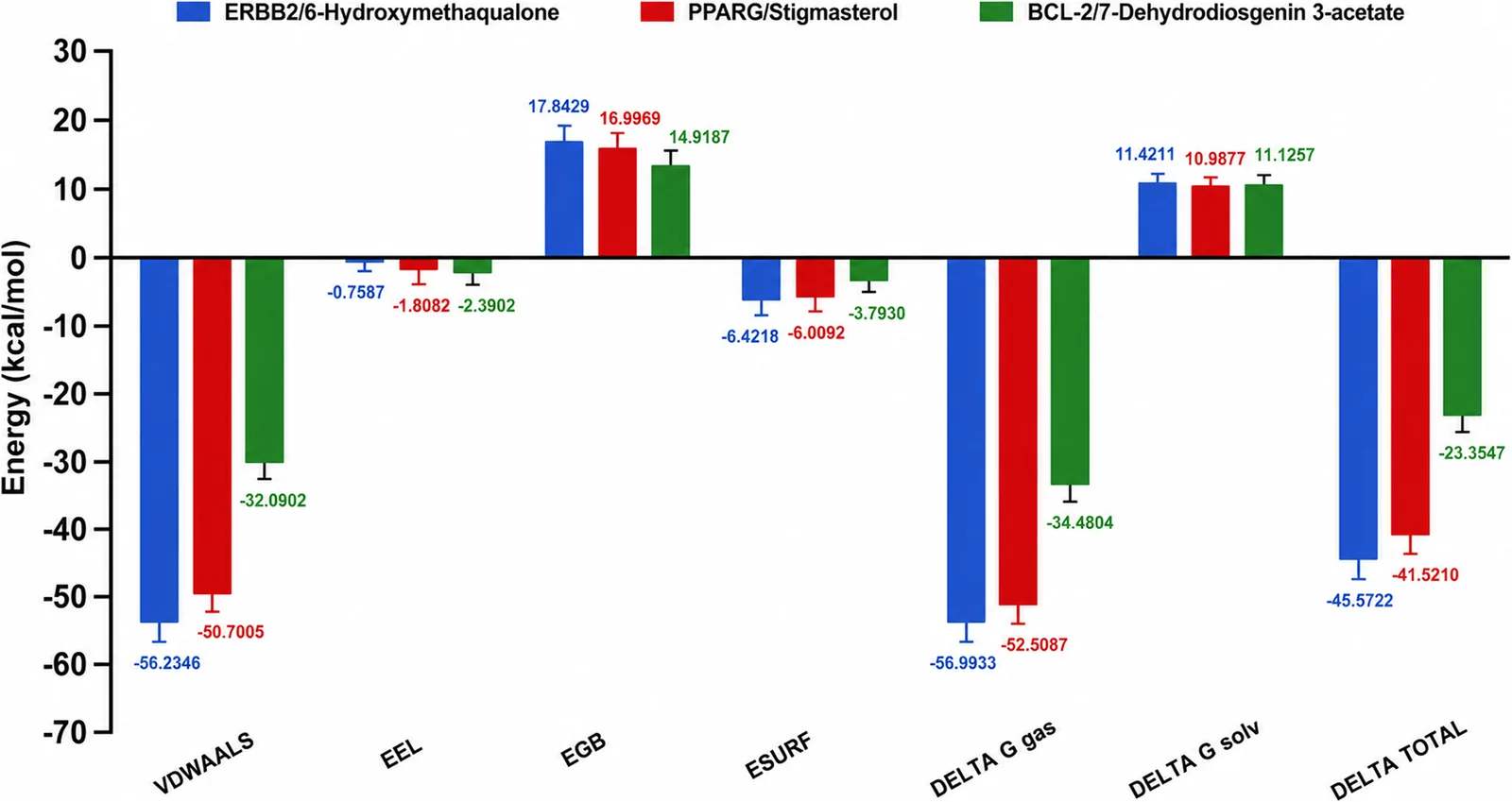
MM/GBSA binding free energy components of the protein–ligand complexes: ERBB2–6-Hydroxymethaqualone (blue), PPARG–Stigmasterol (red), and BCL-2–7-Dehydrodiosgenin 3-acetate (green). All energy values expressed in kcal/mol

The binding free energies of the three selected protein–ligand complexes were further evaluated using the MM/GBSA approach, and the individual energy contributions are summarized in Fig. 12. Among the investigated complexes, the ERBB2–6-Hydroxymethaqualone complex exhibited the most favorable total binding free energy (ΔG_total_ = − 45.57 kcal/mol), followed by PPARG with Stigmasterol (− 41.52 kcal/mol) and BCL2 with 7-Dehydrodiosgenin 3-acetate (− 23.35 kcal/mol). In all three complexes, the binding process was primarily driven by favorable gas-phase interactions (ΔG gas), whereas the solvation free energy (ΔG_solv_) contributed positively, partially offsetting the overall binding affinity.

Van der Waals interactions represented the largest favorable energy component in all systems, with values of − 56.23 kcal/mol for ERBB2–6-Hydroxymethaqualone, − 50.70 kcal/mol for PPARG–Stigmasterol, and − 32.09 kcal/mol for BCL-2–7-Dehydrodiosgenin 3-acetate. In comparison, electrostatic interactions (EEL) contributed only marginally, ranging from − 0.76 to − 2.39 kcal/mol. The gas-phase binding energies (ΔG_gas_) were correspondingly favorable, reaching − 56.99 kcal/mol, − 52.51 kcal/mol, and − 34.48 kcal/mol for the ERBB2, PPARG, and BCL-2 complexes, respectively.

The polar solvation energy (EGB) opposed complex formation in all systems, contributing 17.84 kcal/mol for ERBB2–6-Hydroxymethaqualone, 16.99 kcal/mol for PPARG–Stigmasterol, and 14.92 kcal/mol for BCL-2–7-Dehydrodiosgenin 3-acetate. Conversely, the nonpolar solvation component (E_SURF_) remained favorable, with values of − 6.42, − 6.01, and − 3.79 kcal/mol, respectively. As a result of these opposing solvent contributions, the net solvation free energies (ΔG_solv_) were positive for all complexes, ranging from 10.88 to 11.43 kcal/mol.

### ADMET predictions for *P. piloselloides* compounds

The prediction of ADMET properties is a crucial step in drug development. In this research, three advanced tools (pkCSM, SwissADME, and admetSAR) were employed due to their precision in forecasting the absorption, distribution, metabolism, excretion, and toxicity profiles of drugs and compounds (Table 4). Findings revealed that the intestinal absorption of the test compounds was generally higher than that of the positive control (flutamide).

**Table 4:** Pharmacokinetics and toxicity properties of selected P. piloselloides compounds and positive control

Compound name	No	Absorbtion				Distribution				Metabolisme						
		Human intestinal absorption (%)	Caco2 cell permeability (%)	Caco2 permeability of the query molecule (%)	Skin permeation (Log Kp) (cm/s)	*P*-gp substrate (%)	*P*-gp inhibitor (%)	VDss (human) (log L/Kg)	BBB permeability (%)	CYP 2D6	CYP 3A4	CYP 1A2	CYP 2C19	CYP 2C9	CYP 2D6	CYP 3A4
										Substrate			Inhibitor			
Flutamide (positive control)		87,749	96,20	96,04	-5,61	4,40	30,00	-0,125	95,10	No	Yes	Yes	Yes	No	No	Yes
6-Hydroxymethaqualone	2	97,635	86,01	93,40	-6,40	26,70	16,50	0,007	87,20	No	Yes	Yes	Yes	Yes	No	No
2,6,10,14,18,22-Tetracosahexaene	3	89,002	96,19	88,30	-2,76	14,00	46,20	0,35	93,70	No	Yes	No	No	No	No	No
Ergost-8(14)-en-3-ol, (3.beta.)-	4	94,605	83,60	82,97	-2,89	12,40	28,29	0,483	94,70	No	Yes	No	No	No	No	No
Stigmasterol	5	94,97	81,39	88,25	-2,74	15,60	52,60	0,178	92,70	No	Yes	No	No	No	No	No
Stigmast-7-en-3-ol, (3.beta.,5.alpha.,24 S)-	6	94,464	83,50	87,69	-2,38	12,20	41,10	0,193	93,80	No	Yes	No	No	No	No	No
Stigmastan-3,5,22-trien	7	96,654	84,00	89,32	-1,76	12,50	53,20	0,375	93,00	No	Yes	No	No	No	No	No
7-Dehydrodiosgenin 3-acetate	8	98,825	85,10	82,15	-5,05	20,80	65,30	0,311	96,40	No	Yes	No	No	No	No	No
.beta.-Sitosterol acetate	9	96,576	84,50	86,84	-2,05	12,50	49,50	0,066	94,20	No	Yes	No	No	No	No	No

Excretion							Toxicity									
Total Clearance (log mL/min/kg)	Max. tolerated dose (human) (log mg/kg/day)	AMES toxicity	Carcinogen (%)					Hepato toxicity	Tetrahymena Pyriformis Toxicity (%)		Acute Oral Toxicity (%)		Oral Rat Acute Toxicity (LD50) (mol/kg)		Oral Rat Chronic Toxicity (LOAEL) (log mg/kg)	
			Mouse carcinogenicity (+)	Mouse carcinogenicity (TD50)	Rat carcinogenicity (+)	Rat carcinogenicity (TD50)	Rodents carcinogenicity (+)		TPT (+)	TPT pIG50	AOT (+)	AOT (TD50)				
0,059	0,083	No	48,20	40,79	46,20	37,72	45,50	No	45,50	71,35	71,30	31,35	2,640		1,500	
0,577	-0,007	Yes	37,90	42,14	41,80	23,44	47,00	No	96,00	53,85	87,30	31,98	2,134		1,562	
1,791	-0,533	No	47,90	46,49	37,00	20,69	43,00	No	97,30	91,05	3,00	11,68	1,893		0,911	
0,57	-0,312	No	34,00	39,21	22,50	21,31	25,50	No	97,50	88,68	14,70	20,30	2,038		0,923	
0,618	-0,664	No	38,60	46,07	24,20	24,48	31,80	No	96,00	94,77	8,80	22,19	2,540		0,872	
0,621	-0,621	No	35,90	36,81	23,10	17,08	27,40	No	96,40	89,36	8,50	13,90	2,552		0,855	
0,620	-0,318	No	42,20	41,89	28,39	18,37	36,20	No	96,50	93,28	3,80	8,85	2,171		0,979	
0,311	-0,61	No	44,40	46,85	35,80	32,50	48,10	No	99,30	97,02	2,10	7,42	1,874		1,284	
0,551	-0,476	No	36,50	33,43	23,60	13,16	29,80	No	95,00	89,75	2,80	8,75	2,333		2,461	

The test compounds exhibited a stronger potential to act as P-gp substrates compared to the control compounds. Regarding P-gp inhibition, 6-Hydroxymethaqualone and Ergost-8(14)-en-3-ol, (3.beta.) showed reduced effectiveness relative to the positive control. Additionally, the steady-state volume of distribution (VDss) was lower for all test compounds when compared to the positive control. Regarding Blood-Brain Barrier (BBB) permeability, only 7-Dehydrodiosgenin 3-acetate surpassed the performance of the positive control.

Most test compounds were metabolised by CYP3A4. Conversely, 6-Hydroxymethaqualone was the only compound capable of inhibiting CYP1A2-, CYP2C19-, and CYP2C9-mediated metabolism. The total clearance rates of all test compounds exceeded that of the positive control, with 2,6,10,14,18,22-Tetracosahexaene being the fastest excreted compound among those evaluated.

With regard to toxicity, all test compounds had lower maximum tolerated doses than the positive control. In AMES testing, all compounds were deemed non-mutagenic except for 6-Hydroxymethaqualone. Carcinogenic potential varied across the compounds, although none were predicted to be hepatotoxic.

In terms of Tetrahymena pyriformis toxicity (TPT), most test compounds exhibited greater toxicity than the positive control. Acute oral toxicity was generally lower than the positive control, except in the case of 6-Hydroxymethaqualone. Additionally, all compounds demonstrated reduced Oral Rat Acute Toxicity values relative to the positive control. Under chronic exposure, 6-Hydroxymethaqualone and β-Sitosterol acetate showed higher toxicity than the positive control.

## Discussion

*P. piloselloides* has emerged as a promising source of bioactive phytochemicals with reported anticancer activities against several cancer cell lines, including HeLa and MCF-7 [8, 9]. Previous phytochemical studies also suggested that its phenolic and flavonoid constituents may modulate androgen receptor signaling and epigenetic regulation associated with prostate cancer progression [10, 11]. The present study is based entirely on in silico analyses; therefore, the identified targets, pathways, and compound-protein interactions should be interpreted as hypothesis-generating predictions rather than confirmed mechanisms or therapeutic effects. The present study integrated network pharmacology, molecular docking, molecular dynamics (MD) simulations, and MM/GBSA calculations to identify potential molecular targets and characterize the interactions of *P. piloselloides*-derived compounds with prostate cancer-associated proteins.

Drug-likeness screening indicated that eight compounds satisfied Lipinski’s rule of five, suggesting favorable physicochemical properties for oral drug development [22, 23]. Network pharmacology identified ESR1, MYC, CTNNB1, PPARG, AKT1, STAT3, BCL2, AR, and ERBB2 as hub proteins based on their consistent ranking across multiple topological parameters. Although hub status alone does not establish therapeutic relevance, the biological importance of these proteins is supported by extensive evidence implicating them in prostate cancer initiation, progression, and therapeutic resistance. For example, AR remains the principal driver of prostate cancer development, while AKT1 is a central regulator of the PI3K/AKT signaling pathway that promotes tumor growth and resistance, particularly in PTEN-deficient tumors. Similarly, ERBB2 contributes to aberrant receptor tyrosine kinase signaling associated with aggressive disease, whereas MYC, STAT3, CTNNB1, and BCL2 regulate cell proliferation, survival, stemness, apoptosis, and metastatic progression. In addition, PPARG and ESR1 have been implicated in metabolic reprogramming and hormone-responsive signaling, both of which influence prostate cancer progression and treatment response [16, 38, 39–45].

Functional enrichment analysis further supported the biological relevance of these hub proteins. Gene Ontology enrichment highlighted transcriptional regulation, chromatin organization, and kinase-associated activities, whereas KEGG analysis identified significant enrichment of the PI3K–Akt, ERBB, androgen receptor, and Wnt/β-catenin signaling pathways. These pathways are extensively implicated in prostate cancer biology and collectively regulate cell proliferation, survival, apoptosis, metastasis, and therapeutic resistance, suggesting that *P. piloselloides* compounds may exert multitarget regulatory effects rather than acting through a single molecular mechanism [33, 46–53].

Molecular docking, followed by MD simulations and MM/GBSA calculations, provided complementary computational evidence supporting the predicted compound–target interactions. Among the evaluated phytochemicals, 7-Dehydrodiosgenin 3-acetate demonstrated favorable interactions with MYC, CTNNB1, AKT1, STAT3, BCL2, and ERBB2, while Stigmasterol and 6-Hydroxymethaqualone showed preferential binding toward PPARG and ESR1, respectively. Moreover, several predicted binding affinities for ERBB2, PPARG, and AKT1 were more favorable than those of the corresponding reference ligands within the computational models employed. Although these findings do not confirm biological activity, they provide additional computational support for prioritizing these proteins and compounds for subsequent experimental investigation [54–56].

MD simulations demonstrated that all selected protein-ligand complexes remained structurally stable throughout the 100 ns simulations, with stable RMSD profiles, limited residue fluctuations, and minimal changes in protein compactness, indicating that ligand binding did not substantially disrupt overall protein structures [57]. MM/GBSA analysis further revealed that the ERBB2–6-Hydroxymethaqualone complex exhibited the most favorable binding free energy (ΔG = − 45.57 kcal/mol), followed by PPARG–Stigmasterol (− 41.52 kcal/mol) and BCL2–7-Dehydrodiosgenin 3-acetate (− 23.35 kcal/mol). Van der Waals interactions represented the major energetic contribution, consistent with the predominantly hydrophobic nature of these phytochemicals [58]. Interestingly, the structurally most stable complex was not necessarily associated with the lowest binding free energy, highlighting that conformational stability and binding affinity represent complementary but distinct aspects of protein-ligand interactions [57, 58].

Furthermore, the predicted ADMET profiles of the selected *P. piloselloides* compounds were interpreted in the context of clinically established anticancer agents to provide a pharmacokinetic perspective on their potential drug-like characteristics. Most phytochemicals exhibited high predicted human intestinal absorption (> 89%), exceeding that of the reference compound Flutamide and approaching the efficient oral absorption reported for Enzalutamide (approximately 84% absorbed after oral administration). In contrast, Abiraterone acetate is known to exhibit low and food-dependent oral bioavailability because of its poor solubility and permeability. Although these comparisons involve computational predictions rather than experimental pharmacokinetic measurements, they suggest that several phytochemicals possess physicochemical properties compatible with favorable oral absorption [59–69].

Among the evaluated compounds, 7-Dehydrodiosgenin 3-acetate demonstrated relatively high predicted tissue distribution, P-gp inhibitory activity, and blood-brain barrier permeability. These characteristics may indicate broader systemic distribution; however, they should not be interpreted as evidence of therapeutic efficacy in specific tissues, including the central nervous system, without appropriate experimental validation [61–63].

Most compounds were predicted to be CYP3A4 substrates, indicating that hepatic metabolism through CYP3A4 may represent a common metabolic pathway, similar to that reported for several clinically used anticancer agents, including Enzalutamide, Abiraterone acetate, Docetaxel, and Cabazitaxel. In addition, the absence of predicted inhibition toward major CYP isoforms for most compounds suggests a potentially lower likelihood of CYP-mediated drug-drug interactions, although this prediction requires experimental verification because enzyme induction and inhibition cannot be reliably inferred from in silico analysis alone [60, 64–66].

The predicted toxicity profiles further indicated that all selected compounds were non-hepatotoxic, while only 6-Hydroxymethaqualone showed positive AMES mutagenicity together with broad CYP inhibition, suggesting that this compound may require additional toxicological evaluation. Nevertheless, several phytochemicals displayed lower predicted maximum tolerated doses and higher Tetrahymena pyriformis toxicity than the reference compound, indicating that favorable absorption and metabolic properties should not be interpreted as evidence of an overall superior safety profile. These findings underscore the importance of considering pharmacokinetic and toxicity parameters collectively during compound prioritization [55, 67, 69].

Despite the comprehensive computational workflow employed in this study, the findings should be interpreted within the inherent limitations of in silico approaches. Network pharmacology, molecular docking, molecular dynamics (MD) simulations, MM/GBSA calculations, and ADMET prediction provide complementary insights into compound–target interactions and pharmacokinetic properties but cannot fully capture the complexity of biological systems. In addition, target identification relied on SwissTargetPrediction and PharmMapper, which use different algorithms and reference datasets and may therefore generate false-positive or false-negative predictions. Although predefined filtering criteria were applied before integrating the predicted targets, network pharmacology remains dependent on existing databases and may favor well-characterized proteins while overlooking biologically relevant but less-studied targets. Likewise, MM/GBSA binding free energies should be interpreted as relative computational estimates because explicit entropic contributions were not considered. Therefore, these computational approaches are best regarded as tools for hypothesis generation and candidate prioritization rather than definitive evidence of therapeutic efficacy. Accordingly, the identified phytochemicals should be considered promising candidates for further investigation rather than confirmed therapeutic agents. Although integrating multiple computational approaches provides complementary evidence for candidate prioritization, experimental validation remains essential to confirm target engagement, biological activity, pharmacokinetic behavior, and safety. Future studies combining experimental validation with advanced free-energy calculations and structure–activity relationship optimization would further strengthen the translational relevance of these findings.

## Conclusion

In conclusion, the integrated computational analysis identified multiple prostate cancer–related targets associated with *P. piloselloides* compounds, with ESR1, MYC, CTNNB1, PPARG, AKT1, STAT3, BCL2, AR, and ERBB2 emerging as core targets. These multi-target associations were linked to enrichment of diverse biological processes and 161 signaling pathways relevant to prostate cancer, including the prostate cancer and PI3K–Akt signaling pathways. Molecular docking identified 7-dehydrodiosgenin 3-acetate, stigmasterol, and 6-hydroxymethaqualone as compounds with favorable predicted binding affinities across several key targets. Molecular dynamics simulations indicated overall structural stability with localized conformational flexibility, while MM/GBSA analysis yielded favorable predicted binding free energies that consistently highlighted these three compounds as the most favorable candidates among the evaluated interactions. The *P. piloselloides* compounds also showed generally favorable predicted ADMET profiles, providing a basis for further investigation and supporting their prioritization for experimental evaluation. These findings may help guide the selection of compounds and molecular targets for subsequent experimental studies of their potential relevance to prostate cancer. 

## Supplementary Information

Supplementary Material 1.

## Data Availability

No datasets were generated or analysed during the current study.
