# Supplementary material for: Computational network pharmacology and molecular simulations to explore the potential mechanisms of Pyrrosia piloselloides compounds against prostate cancer
Source: J Egypt Natl Canc Inst. 2026 Oct 29;38:83. doi: 10.1186/s43046-026-00421-3 (PMC13719924; doi:10.1186/s43046-026-00421-3)
Supplement: Supplementary file 1 [file 43046_2026_421_MOESM1_ESM.docx]

**Computational Network Pharmacology and Molecular Simulations to Explore the Potential Mechanisms of *Pyrrosia piloselloides* Compounds Against Prostate Cancer**

**Table S1.** Structural information, optimized molecular docking parameters (grid box coordinates, dimensions, and exhaustiveness), and RMSD values obtained from redocking validation for the selected hub proteins.

| Protein | PDB ID | Grid Spacing | Center | | | Size (X×Y×Z) | Exhaustiveness | RMSD validation |
| --- | --- | --- | --- | --- | --- | --- | --- | --- |
|  |  |  | X | Y | Z |  |  |  |
| ESR1 | 2IOK | 0.375 | 17.608 | 35.449 | 52.619 | 40x40x40 | 20 | <2 |
| MYC | 1NKP |  | 74.25 | 77.055 | 39.187 | 40x80x52 |  |  |
| CTNNB1 | 7AFW |  | 59.556 | -40.342 | 17.8 | 23x10.5x11 |  |  |
| PPARG | 2PRG |  | 49.889 | -37.024 | 19.274 | 40x40x40 |  |  |
| AKT1 | 4GV1 |  | -20.345 | 4.713 | 10.896 | 40x40x40 |  |  |
| STAT3 | 6NJS |  | 13.498 | 54.117 | 0.1 | 11.20x20.80x12 |  |  |
| BCL-2 | 4IEH |  | 12.435 | 25.124 | 11.034 | 40x40x40 |  |  |
| AR | 2AX9 |  |  |  |  |  |  |  |
| ERBB2 | 3PP0 |  | 34.801 | 44.589 | -11.371 | 40x40x40 |  |  |
